# Supplementary material for: Transcriptome-wide profiles of circular RNA and RNA-binding protein interactions reveal effects on circular RNA biogenesis and cancer pathway expression
Source: Genome Med. 2020 Dec 7;12:112. doi: 10.1186/s13073-020-00812-8 (PMC7722315; doi:10.1186/s13073-020-00812-8)
Supplement: Supplementary file 8 — Additional file 8: Fig. S1-S6. Pdf file with all supplementary figures (Fig. S1-S6) with corresponding figure legends. [file 13073_2020_812_MOESM8_ESM.pdf]

Fig. S1

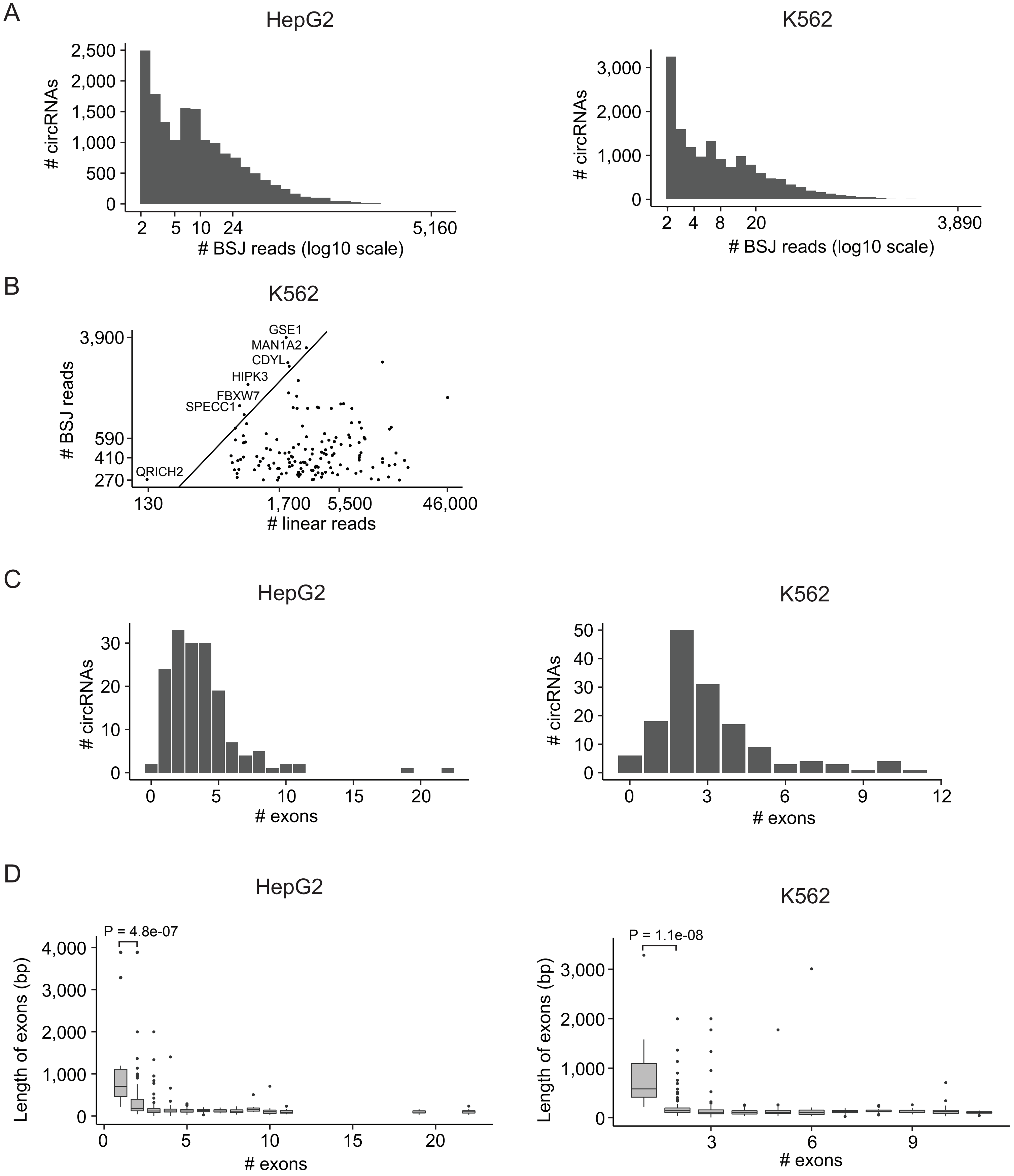
A
HepG2
circRNAs
BSJ reads (log10 scale)
K562
circRNAs
BSJ reads (log10 scale)
B
K562
BSJ reads
linear reads
GSE1
MAN1A2
CDYL
HIPK3
FBXW7
SPECC1
QRICH2
C
HepG2
circRNAs
exons
K562
circRNAs
exons
D
HepG2
P = 4.8e-07
Length of exons (bp)
exons
K562
P = 1.1e-08
Length of exons (bp)
exons

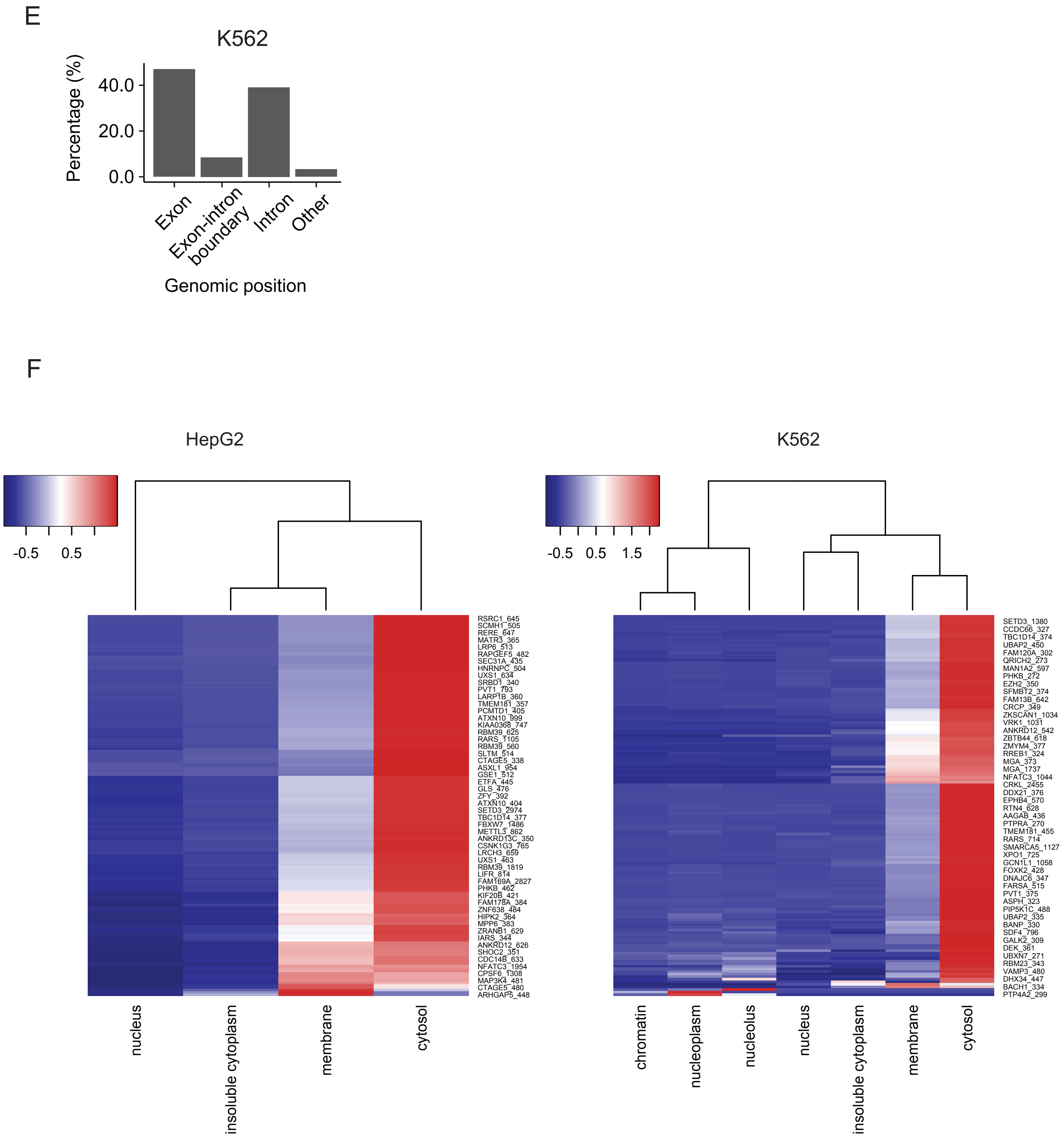

Fig. S1: Circular RNAs are highly expressed in HepG2 and K562 and generally colocalizes with RBPs
A) Number of backsplice junction (BSJ) spanning reads supporting all circRNAs in HepG2 (left, n = 16,033) and K562 (right, n = 14,648). X-axis is plotted on a logarithmic scale (log10). **B)** Number of backsplice junction (BSJ) reads supporting the 1% highest expressed circRNAs (n = 147) and the number of reads spanning canonical splice sites in the corresponding linear transcripts in K562. X- and Y-axis are plotted on a logarithmic scale (log10) showing actual counts. Some circRNAs are depicted with their host gene name. The number of reads represent the summarized values across all K562 data sets. **C)** Number of exons comprising highly expressed circRNAs in HepG2 (left) and K562 (right). 74% (HepG2) and 83% (K562) of the top 1% expressed circRNAs contain less than 5 exons, while 15% (HepG2) and 12% (K562) of the circRNAs originate from just one exon. The x-axis shows the number of exons within the circRNA loci. **D)** Length of exons comprising the top 1% circRNAs in HepG2 (left) and K562 (right). The x-axis shows the number of exons within the circRNA loci. **E)** Genomic location of RBP binding sites in the top 1% circRNA loci in K562. Most RBP binding sites are found within exonic parts of the circRNAs. **F)** Heatmap of circRNA expression (top 1 %) in subcellular fractions of HepG2 (left, n = 4) and K562 (right, n = 7).

Fig. S2

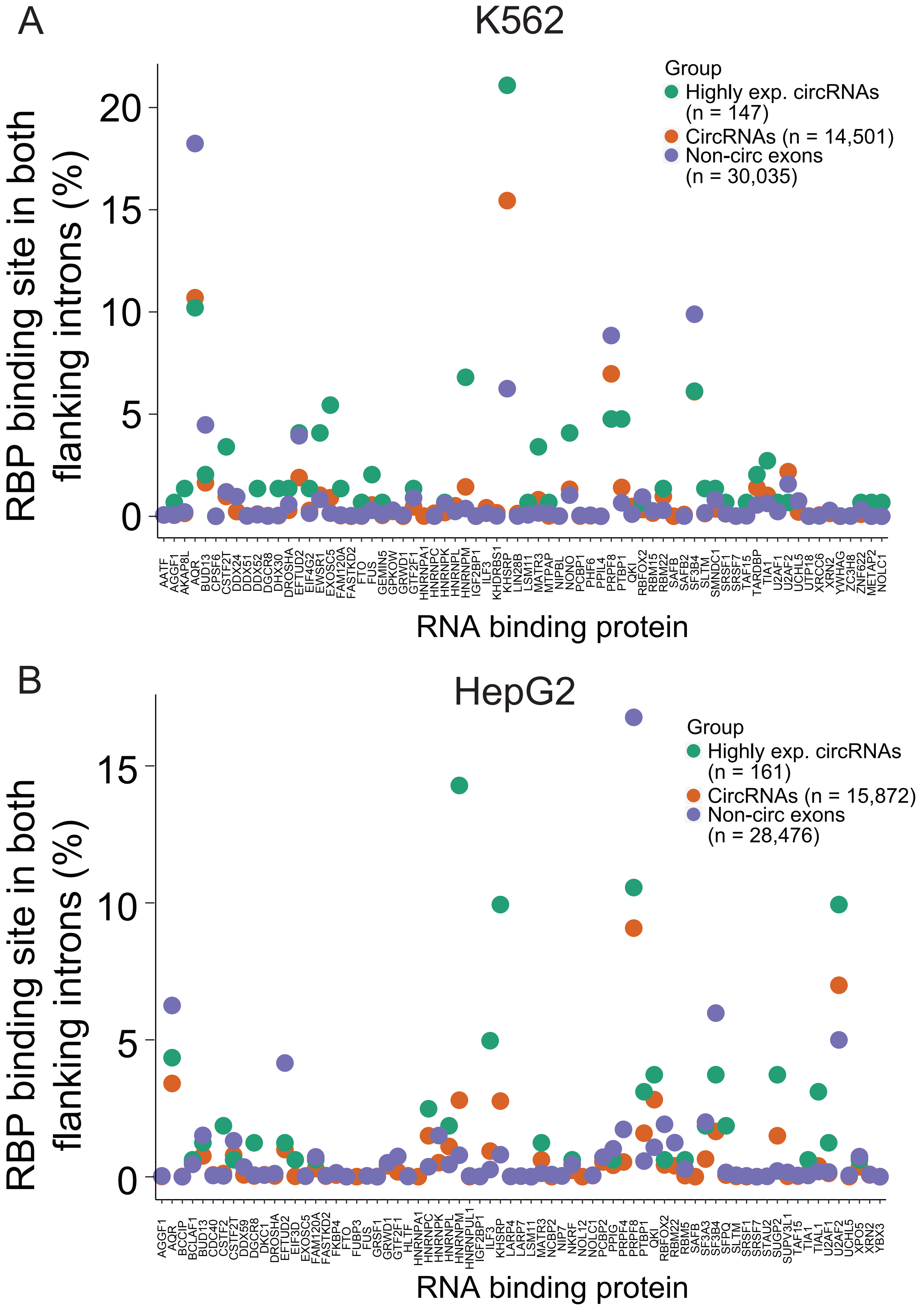
A
K562
RBP binding site in both flanking introns (%)
0
5
10
15
20
Group
Highly exp. circRNAs (n = 147)
CircRNAs (n = 14,501)
Non-circ exons (n = 30,035)
AATF AGGF1 AKAP8L AQR BUD13 CPSF6 CSTF2T DDX24 DDX51 DDX52 DGCR8 DHX30 DROSHA EFTUD2 EIF4G2 EWSR1 EXOSC5 FAM120A FASTKD2 FTO FUS GEMIN5 GPKOW GRWD1 GTF2F1 HNRNPA1 HNRNPC HNRNPK HNRNPL HNRNPM IGF2BP1 ILF3 KHDRBS1 KHSRP LIN28B LSM11 MATR3 MTPAP NIPBL NONO PCBP1 PHF6 PPIL4 PRPF8 PTBP1 QKI RBFOX2 RBM15 RBM22 SAFB SAFB2 SF3B4 SLTM SMNDC1 SRSF1 SRSF7 TAF15 TARDBP TIA1 U2AF1 U2AF2 UCHL5 UTP18 XRCC6 XRN2 YWHAG ZC3H8 ZNF622 METAP2 NOLC1
RNA binding protein
B
HepG2
RBP binding site in both flanking introns (%)
0
5
10
15
Group
Highly exp. circRNAs (n = 161)
CircRNAs (n = 15,872)
Non-circ exons (n = 28,476)
AGGF1 AQR BCCIP BCLAF1 BUD13 CDC40 CSTF2 CSTF2T DDX59 DGCR8 DKC1 DROSHA EFTUD2 EIF3D EXOSC5 FAM120A FASTKD2 FKBP4 FTO FUBP3 FUS GRSF1 GRWD1 GTF2F1 HLTF HNRNPA1 HNRNPC HNRNPK HNRNPL HNRNPM HNRNPUL1 IGF2BP1 ILF3 KHSRP LARP4 LARP7 LSM11 MATR3 NCBP2 NIP7 NKRF NOL12 NOLC1 PCBP2 PPIG PRPF4 PRPF8 PTBP1 QKI RBFOX2 RBM22 RBM5 SAFB SF3A3 SF3B4 SFPQ SLTM SRSF1 SRSF7 STAU2 SUGP2 SUPV3L1 TAF15 TIA1 TIAL1 U2AF1 U2AF2 UCHL5 XPO5 XRN2 YBX3
RNA binding protein

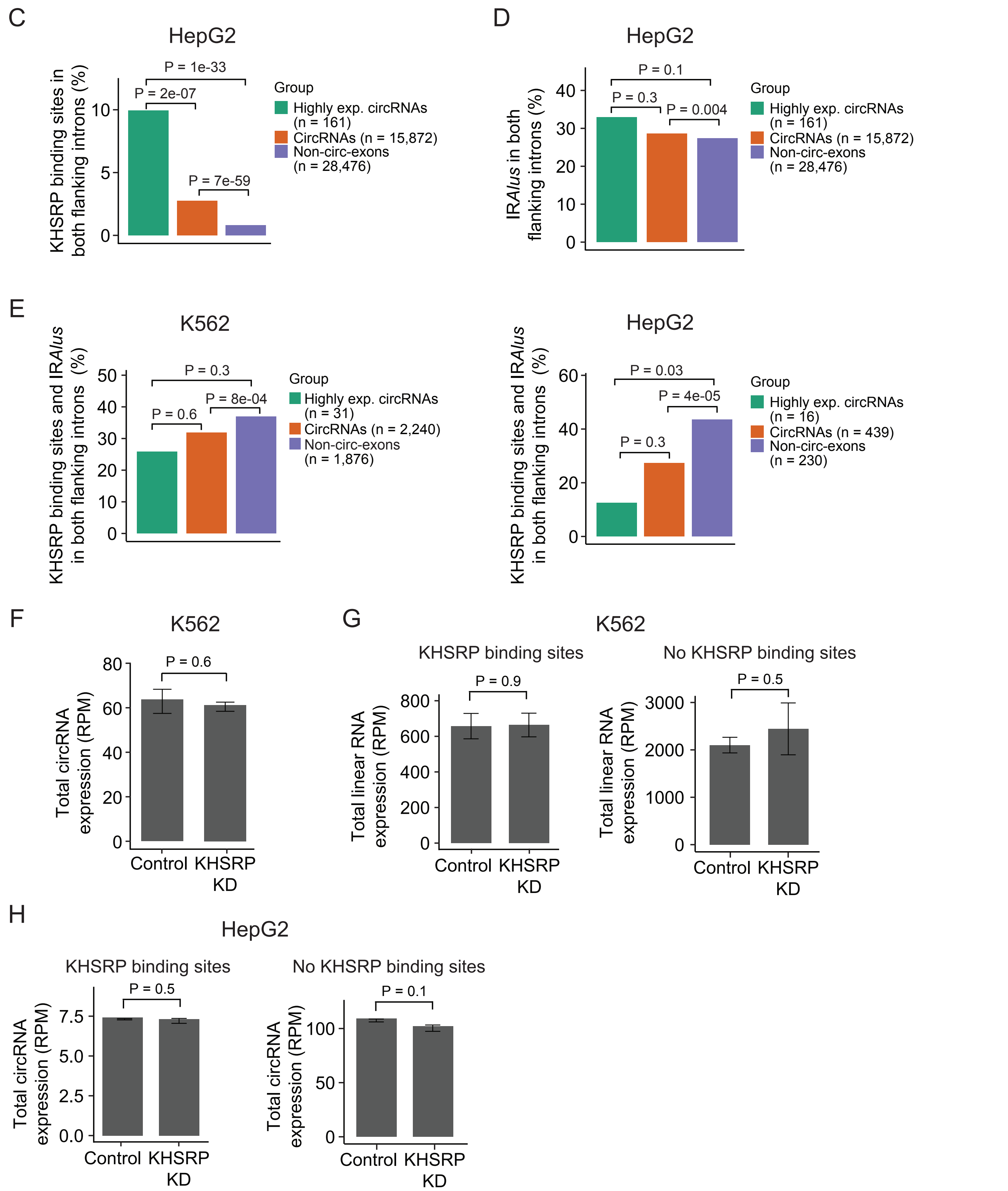

Fig. S2: KHSRP binding is enriched in introns flanking circRNAs and affects biogenesis
A+B) Percentage of circRNAs and non-circ-exons with RBP binding sites in both flanking introns for each RBP in K562 (A) and HepG2 (B). **C)** Percentage of circRNAs and non-circ-exons in genes producing circRNAs with KHSRP binding sites in both flanking introns in HepG2. P-values obtained by Chi-square Test. **D)** Percentage of circRNAs and non-circ-exons in genes producing circRNAs with inverted repeated *Alu* elements (IR*Alus*) in both flanking introns in HepG2. P-values obtained by Chi-square Test. **E)** Percentage of circRNAs and non-circ-exons in genes producing circRNAs with KHSRP binding sites in both flanking introns that are also surrounded by IR*Alus* in K562 (left) and HepG2 (right). P-values obtained by Chi-square Test. **F)** Total circRNA expression (n = 1,834) in KHSRP knockdown (KD) and control samples in K562. P-values obtained by T-test. RPM = reads per million. **G)** Total expression of the corresponding linear RNAs of circRNAs with (left, n = 297) and without (right, n = 1,537) KHSRP binding sites in both flanking introns in KHSRP knockdown (KD) and control samples in K562. P-values obtained by T-test. **H)** Total expression of circRNAs with (left, n = 97) and without (right, n = 2,541) KHSRP binding sites in both flanking introns in KHSRP knockdown (KD) and control samples in HepG2. P-values obtained by T-test.

Fig. S3

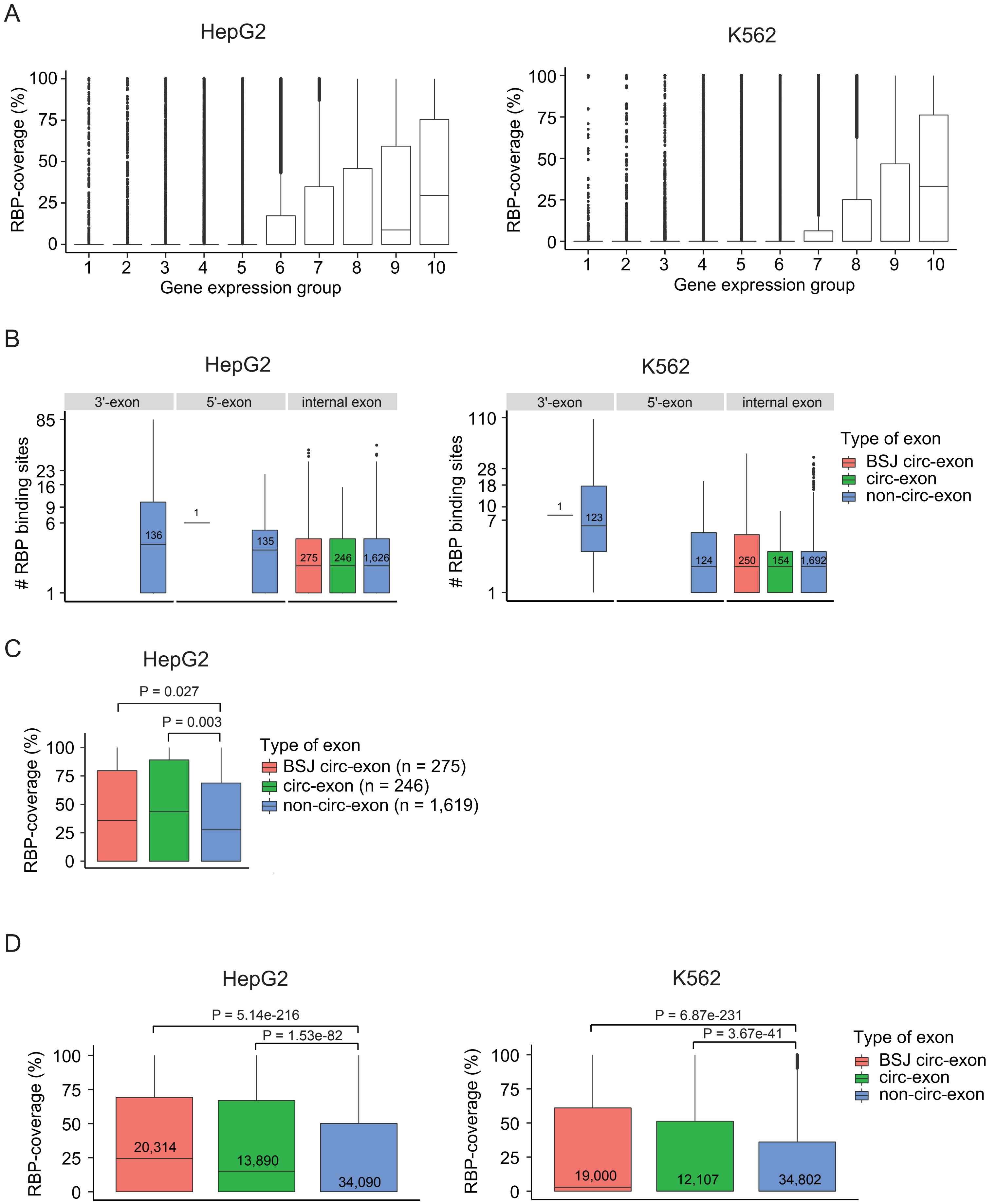
A
HepG2
K562
RBP-coverage (%)
Gene expression group
B
HepG2
K562
3'-exon
5'-exon
internal exon
RBP binding sites
136
1
135
275
246
1,626
123
124
250
154
1,692
Type of exon
BSJ circ-exon
circ-exon
non-circ-exon
C
HepG2
P = 0.027
P = 0.003
RBP-coverage (%)
Type of exon
BSJ circ-exon (n = 275)
circ-exon (n = 246)
non-circ-exon (n = 1,619)
D
HepG2
K562
P = 5.14e-216
P = 1.53e-82
P = 6.87e-231
P = 3.67e-41
RBP-coverage (%)
20,314
13,890
34,090
19,000
12,107
34,802
Type of exon
BSJ circ-exon
circ-exon
non-circ-exon

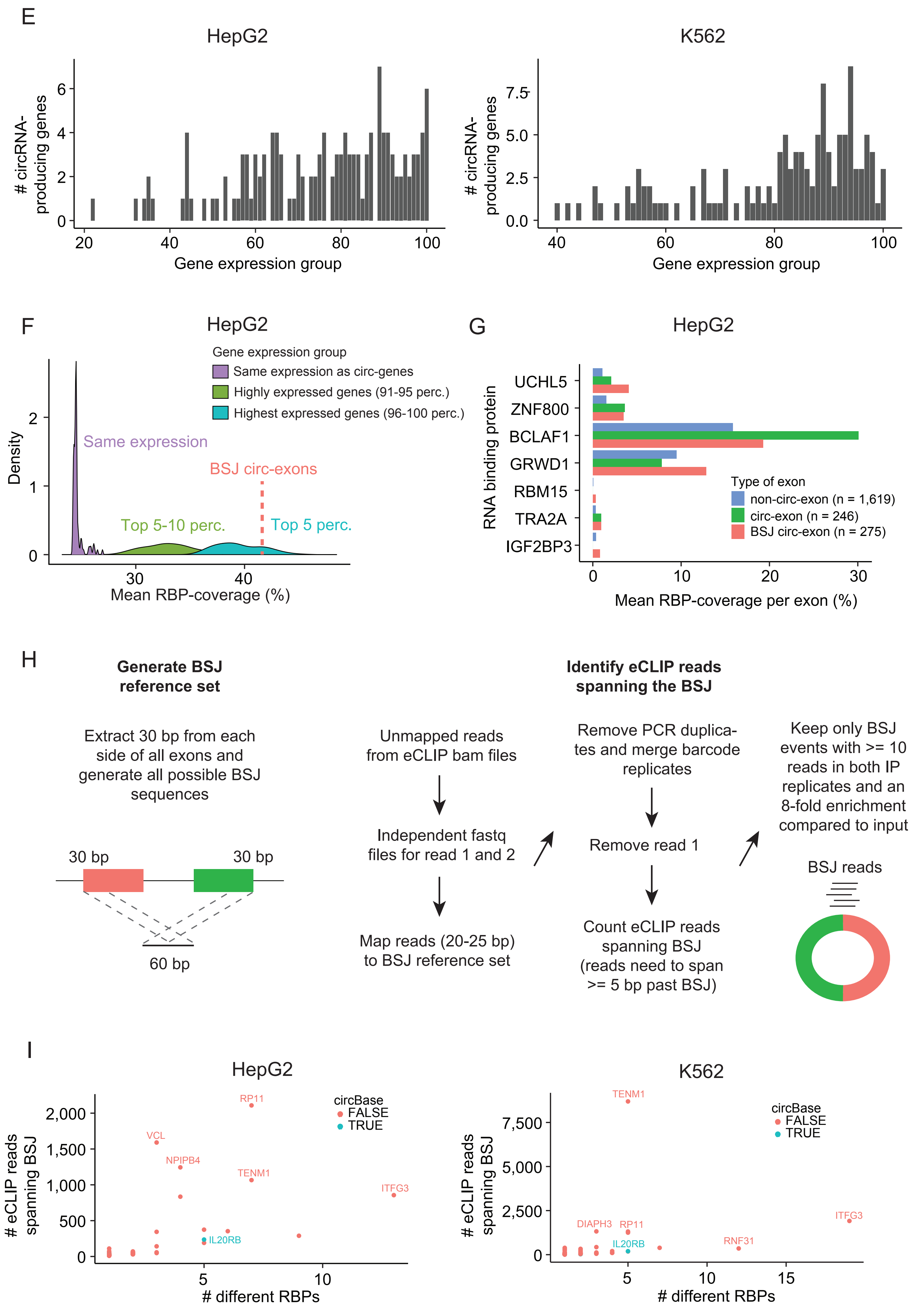
E
HepG2
circRNA-producing genes
Gene expression group
K562
circRNA-producing genes
Gene expression group
F
HepG2
Gene expression group
Same expression as circ-genes
Highly expressed genes (91-95 perc.)
Highest expressed genes (96-100 perc.)
Same expression
BSJ circ-exons
Top 5-10 perc.
Top 5 perc.
Density
Mean RBP-coverage (%)
G
HepG2
RNA binding protein
UCHL5
ZNF800
BCLAF1
GRWD1
RBM15
TRA2A
IGF2BP3
Type of exon
non-circ-exon (n = 1,619)
circ-exon (n = 246)
BSJ circ-exon (n = 275)
Mean RBP-coverage per exon (%)
H
Generate BSJ reference set
Extract 30 bp from each side of all exons and generate all possible BSJ sequences
30 bp
30 bp
60 bp
Unmapped reads from eCLIP bam files
Independent fastq files for read 1 and 2
Map reads (20-25 bp) to BSJ reference set
Identify eCLIP reads spanning the BSJ
Remove PCR duplicates and merge barcode replicates
Remove read 1
Count eCLIP reads spanning BSJ (reads need to span >= 5 bp past BSJ)
Keep only BSJ events with >= 10 reads in both IP replicates and an 8-fold enrichment compared to input
BSJ reads
I
HepG2
eCLIP reads spanning BSJ
different RBPs
circBase
FALSE
TRUE
RP11
VCL
NPIPB4
TENM1
ITFG3
IL20RB
K562
eCLIP reads spanning BSJ
different RBPs
circBase
FALSE
TRUE
TENM1
DIAPH3
RP11
IL20RB
RNF31
ITFG3

Fig. S3: Exons comprising circRNAs are enriched with RBP binding sites

A) Fraction of exons covered with RBP binding sites in different gene expression groups. All expressed protein coding genes in HepG2 (n = 18,152) and K562 (n = 18,784) were divided into ten bins based on expression. RBP-coverage correlates with gene expression in HepG2 (R = 0.41, P < 2.2e-16, Pearson's product-moment correlation) and K562 (R = 0.42, P < 2.2e-16, Pearson's product-moment correlation). **B)** Number of RBP binding sites in 5'-, 3'-, and internal exons of genes that produce highly expressed (top 1%) circRNAs. Numbers represent the number of exons in each group. The y-axis is plotted on a log10-scale but showing real numbers of RBP binding sites. **C)** RBP binding site coverage for each category of exons in genes that produce highly expressed (top 1%) circRNAs in HepG2 (n = 135). P-values obtained by Wilcoxon Rank Sum Test. **D)** RBP-coverage for each category of exons in genes that produce circRNAs (supported by at least two reads) in HepG2 (n = 4,347genes) and K562 (n = 4,389 genes). Numbers represent the number of exons in each group. P-values obtained by Wilcoxon Rank Sum Test. **E)** Gene expression group distribution of genes producing highly expressed circRNAs in HepG2 (n = 135 genes) and K562 (n = 124). In cases where more than one circRNA arises from the same gene, the gene is only counted once. **F)** Comparison of RBP-coverage between BSJ circ-exons and exons in groups of genes of different expression levels (HepG2). The mean RBP-coverage of the highly expressed BSJ circ-exons (n = 275) is 42% (red punctuated line; median = 36%). Exons randomly sampled from genes while ensuring the same expression profile as genes producing highly expressed circRNAs (circ-genes) have a much lower mean coverage of 25% (purple; median = 0%). Exons of highly expressed genes (top 5-10 percentiles) also showed a lower mean coverage of 33% (green; median = 13%), while the most highly expressed genes (top 5 percentiles) had a mean of 40% (blue; median = 29%). The random sampling procedures were repeated with 100 iterations. Empirical P-values for: BSJ circ-exons vs. same expression, P < 0.01; BSJ circ-exons vs. Top5-10, P < 0.01; BSJ circ-exons vs Top5, P = 0.2. **G)** Mean RBP-coverage per exon for individual RBPs in HepG2. All RBPs shown here have significantly more target sites in highly expressed BSJ circ-exons than non-circ-exons of the same genes (FDR < 0.1, Wilcoxon Rank Sum Test). Only RBPs with at least 20 distinct binding sites in total are considered. **H)** Approach for identifying eCLIP reads spanning the backsplice junction (BSJ) of circRNAs. By extracting 30 bp from each side of all exons, we generated a reference set of all possible exonic BSJ events. We extracted all unmapped eCLIP reads and mapped them against the BSJ reference set, allowing two mismatches. Next, we removed PCR duplicates and merged barcode replicates. We removed read 1 (which was only used to identify and remove PCR duplicates) and counted eCLIP reads spanning each BSJ. Only reads spanning a BSJ by at least 5 bp were counted. To identify specific RBP binding sites, we only reported binding sites supported by at least 10 reads in both IP replicates and with an 8-fold enrichment in IP compared to input (Methods). **I)** Number of eCLIP reads spanning BSJ events in HepG2 (left) and K562 (right). Some BSJ events are supported by eCLIP reads, however, none of these correspond to a circRNA predicted by CIRI2.

Fig. S4

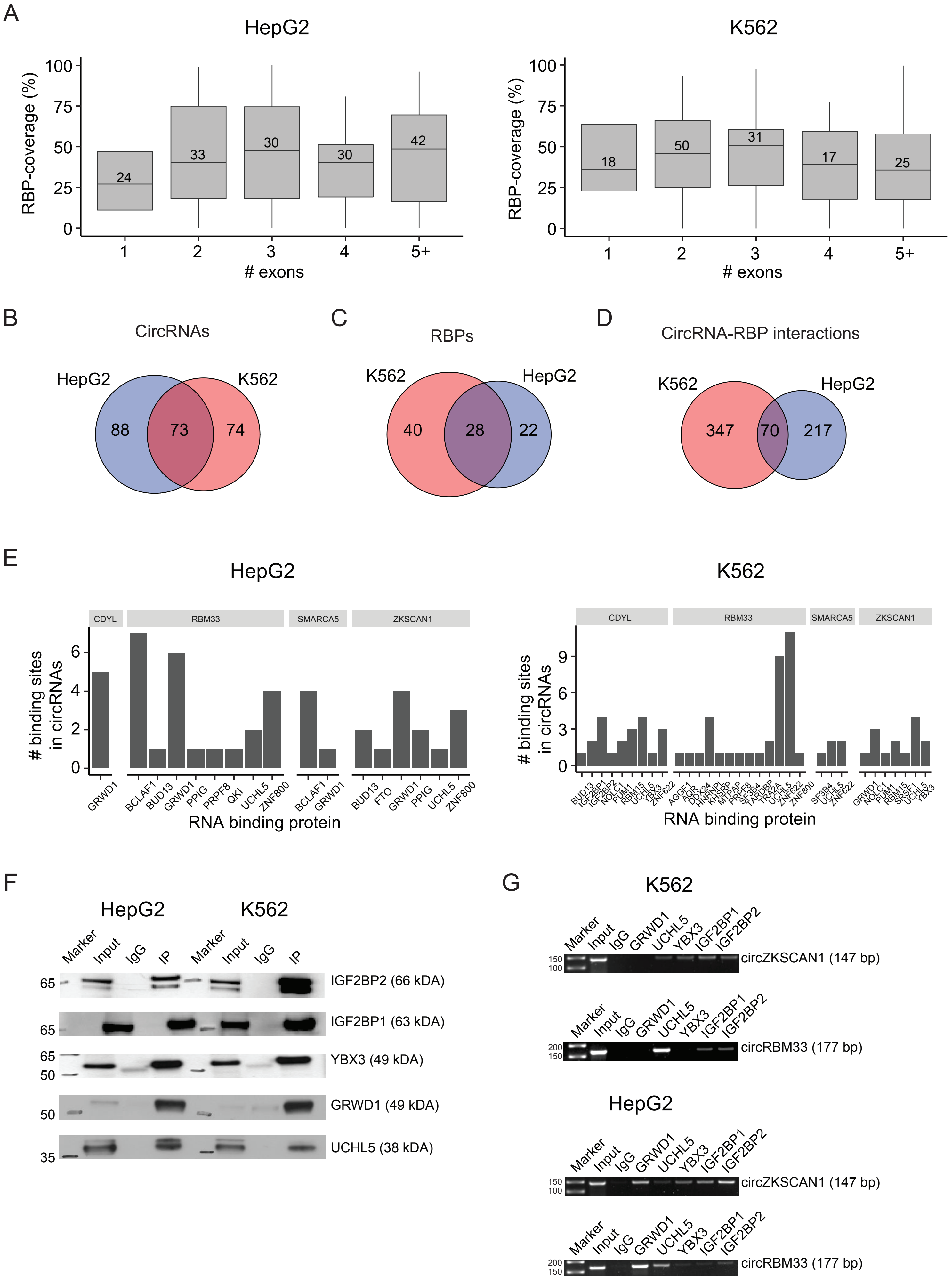
A
HepG2
K562
RBP-coverage (%)
exons
1
2
3
4
5+
0
25
50
75
100
24
33
30
30
42
18
50
31
17
25
B
CircRNAs
HepG2
K562
88
73
74
C
RBPs
K562
HepG2
40
28
22
D
CircRNA-RBP interactions
K562
HepG2
347
70
217
E
HepG2
K562
CDYL
RBM33
SMARCA5
ZKSCAN1
binding sites in circRNAs
RNA binding protein
GRWD1
BCLAF1
BUD13
GRWD1
PPIG
PRPF8
QKI
UCHL5
ZNF800
BCLAF1
GRWD1
BUD13
FTO
GRWD1
PPIG
UCHL5
ZNF800
F
HepG2
K562
Marker
Input
IgG
IP
IGF2BP2 (66 kDA)
IGF2BP1 (63 kDA)
YBX3 (49 kDA)
GRWD1 (49 kDA)
UCHL5 (38 kDA)
65
50
35
G
K562
HepG2
Marker
Input
IgG
GRWD1
UCHL5
YBX3
IGF2BP1
IGF2BP2
circZKSCAN1 (147 bp)
circRBM33 (177 bp)
200
150
100

Fig. S4: CircRNAs interact with RBPs in a cell-type-specific manner

A) RBP-coverage based on the number of exons comprising the top 1% circRNAs in HepG2 (left) and K562 (right). Numbers indicate the number of circRNAs belonging to each group. **B)** Of the 1% highest expressed circRNAs in HepG2 (n = 161) and K562 (n = 147), 73 circRNAs are highly expressed in both cell lines. **C)** Of RBPs with binding sites in the shared highly expressed circRNAs in HepG2 (n = 50) and K562 (n = 68), 28 RBPs are evaluated in both cell lines. **D)** CircRNA-RBP interactions identified in both cell lines. 70 interactions between 73 highly expressed circRNAs and 28 RBPs are identified in both HepG2 and K562. The genomic positions of RBP binding sites are not considered. **E)** Number of predicted binding sites for each RBP in four highly expressed and RBP-covered circRNAs; circCDYL, circRBM33, circSMARCA5, and circZKSCAN1. **F)** Western blot of IP and input from the RIP experiments. IgG is used as negative control. **G)** Validation of circRNA-RBP interactions from RIP experiments. Here, RIP experiments confirmed that circRBM33 interacts strongly with UCHL5 in K562 and with both GRWD1 and UCHL5 in HepG2. For circZKSCAN1, several interactions were observed, however, binding affinities were generally low. Specifically, circZKSCAN1 contains a single binding site for GRWD1 in K562 (Fig. S4E), but no interaction was detected from the RIP experiments. 50 bp markers used.

Fig. S5

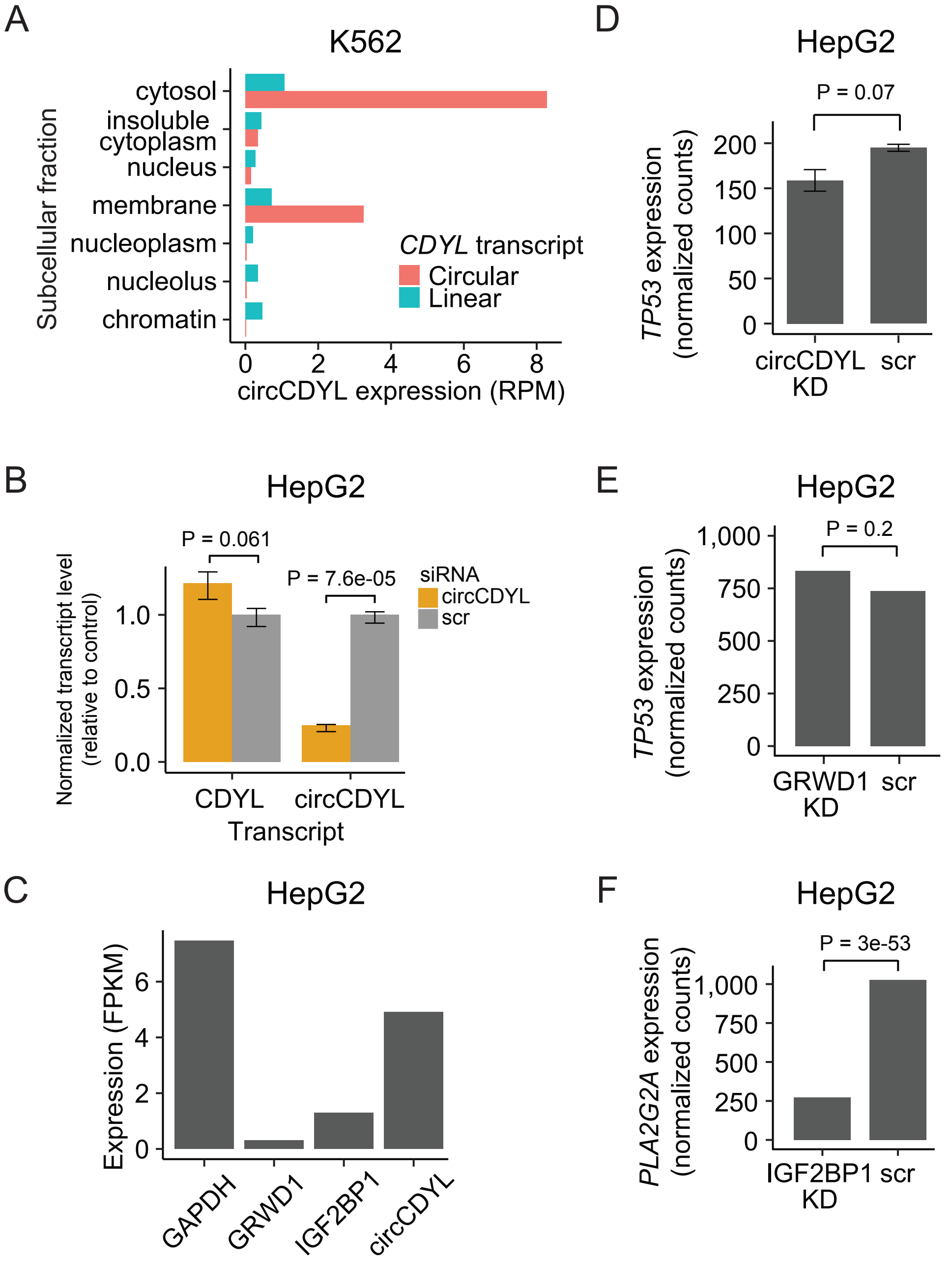

Fig. S5: Functional studies of circCDYL-RBP interactions in HepG2

A) Expression of circCDYL and the corresponding linear transcript in subcellular fractions of K562. **B)** Normalized expression levels of circCDYL and the *CDYL* gene upon circCDYL KD in HepG2 cells. Expression is normalized to *GAPDH* and plotted relative to control (scr samples). P-values obtained by T-test. **C)** Expression of the exon that constitutes circCDYL (chr6:4891946-4892613) and the mRNAs of its interacting RBPs, *GRWD1* and *IGF2BP1*, in HepG2 cells. *GAPDH* is used as reference for a highly expressed gene. **D+E)** Expression of *TP53* upon circCDYL KD (D) and GRWD1 KD (E) in HepG2 cells. P-values obtained by Wald Test. **F)** Expression of *PLA2G2A* upon IGF2BP1 KD in HepG2 cells. P-value obtained by Wald Test.

Fig. S6

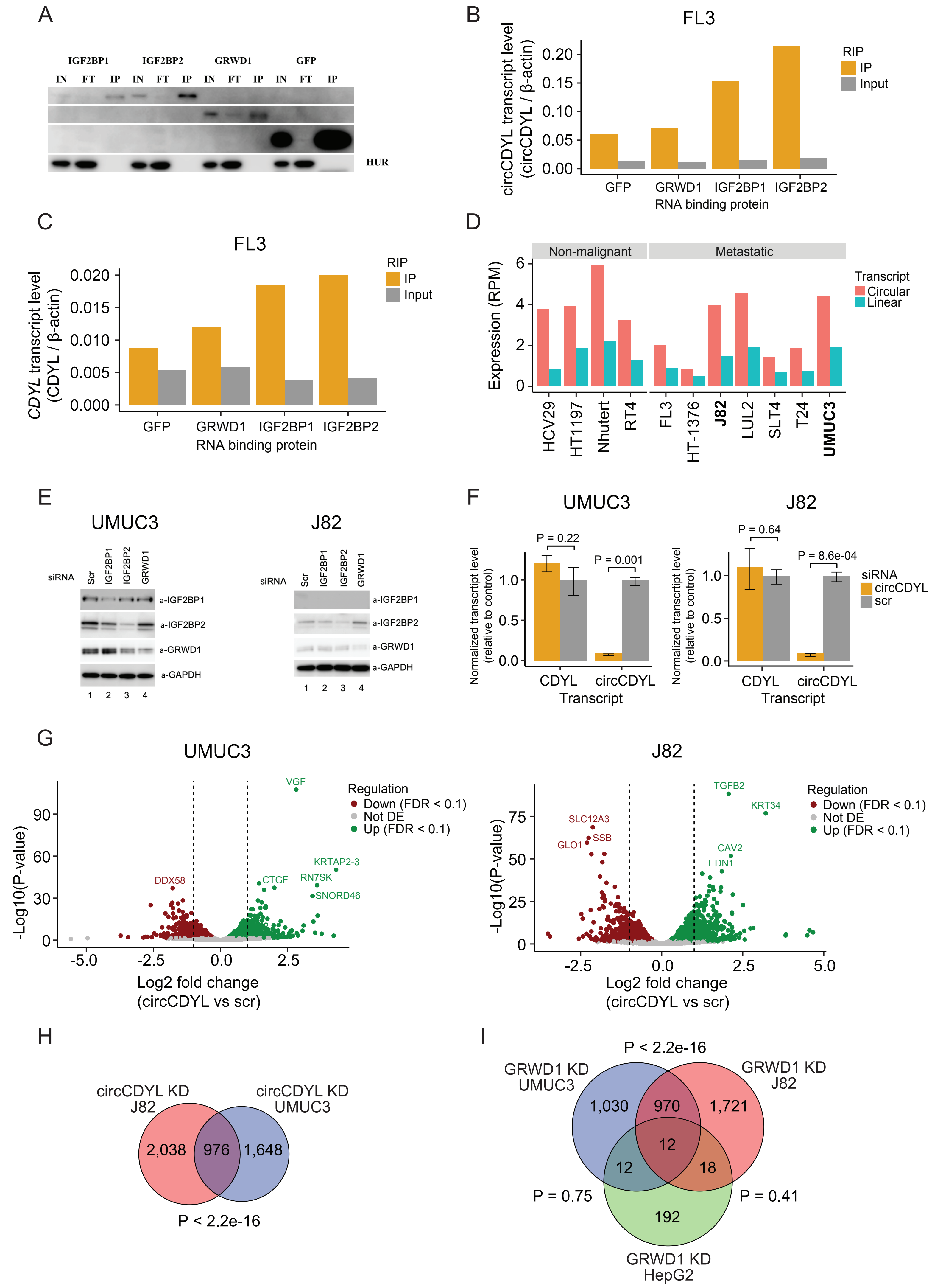
A
IGF2BP1
IGF2BP2
GRWD1
GFP
IN FT IP IN FT IP IN FT IP IN FT IP
HUR
B
FL3
circCDYL transcript level
(circCDYL / β-actin)
RIP
IP
Input
GFP GRWD1 IGF2BP1 IGF2BP2
RNA binding protein
C
FL3
CDYL transcript level
(CDYL / β-actin)
RIP
IP
Input
GFP GRWD1 IGF2BP1 IGF2BP2
RNA binding protein
D
Non-malignant
Metastatic
Expression (RPM)
Transcript
Circular
Linear
HCV29 HT1197 Nhutert RT4 FL3 HT-1376 J82 LUL2 SLT4 T24 UMUC3
E
UMUC3
J82
siRNA
Scr IGF2BP1 IGF2BP2 GRWD1
a-IGF2BP1
a-IGF2BP2
a-GRWD1
a-GAPDH
1 2 3 4
F
UMUC3
J82
P = 0.22
P = 0.001
P = 0.64
P = 8.6e-04
Normalized transcript level
(relative to control)
CDYL circCDYL
Transcript
siRNA
circCDYL
scr
G
UMUC3
J82
-Log10(P-value)
Log2 fold change
(circCDYL vs scr)
Regulation
Down (FDR < 0.1)
Not DE
Up (FDR < 0.1)
VGF
KRTAP2-3
RN7SK
SNORD46
CTGF
DDX58
TGFB2
KRT34
SLC12A3
SSB
GLO1
CAV2
EDN1
H
circCDYL KD
J82
circCDYL KD
UMUC3
2,038
976
1,648
P < 2.2e-16
I
P < 2.2e-16
GRWD1 KD
UMUC3
GRWD1 KD
J82
1,030
970
1,721
12
12
18
192
P = 0.75
P = 0.41
GRWD1 KD
HepG2

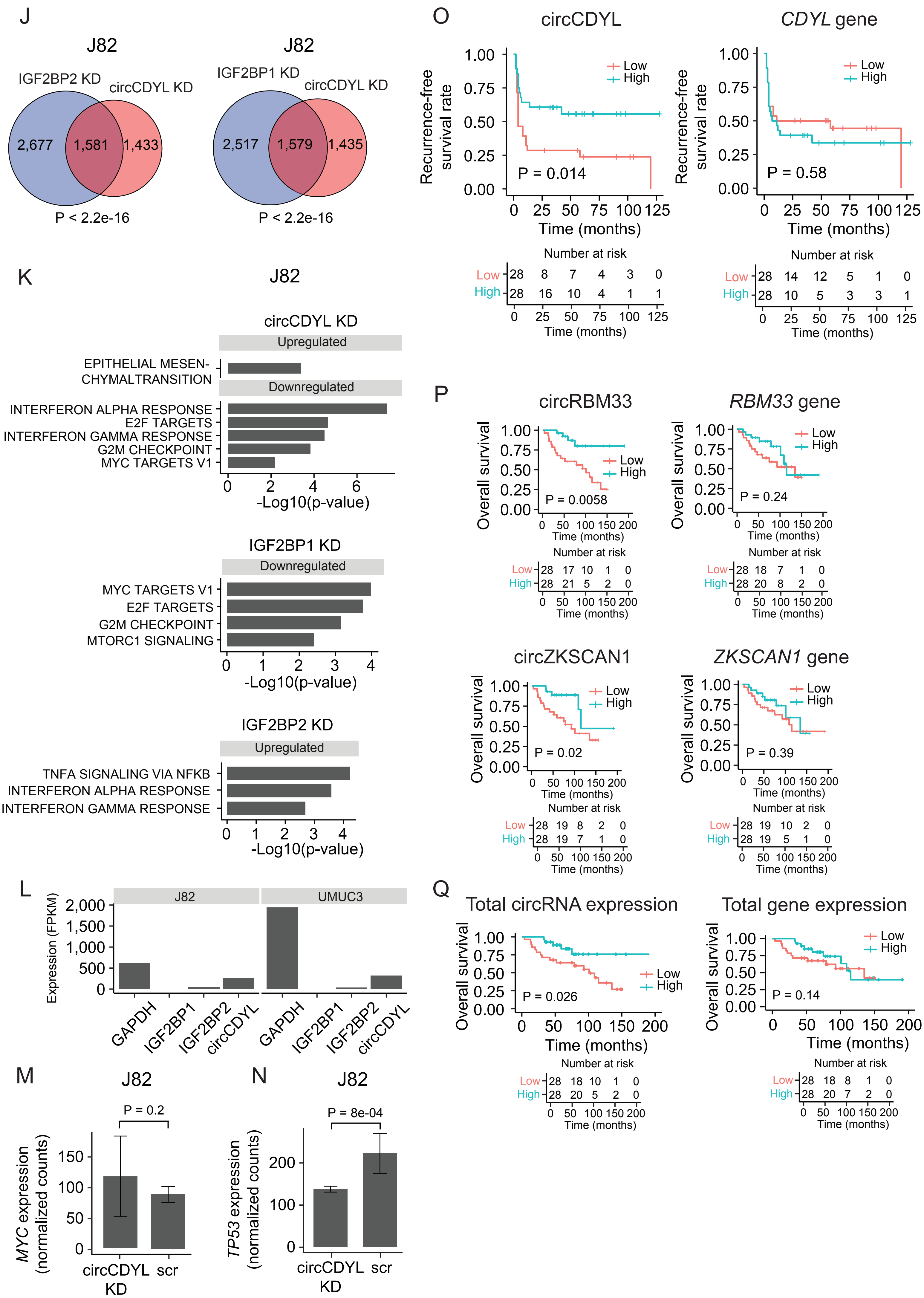
J
J82
IGF2BP2 KD
circCDYL KD
2,677
1,581
1,433
P < 2.2e-16
J82
IGF2BP1 KD
circCDYL KD
2,517
1,579
1,435
P < 2.2e-16
O
circCDYL
Recurrence-free survival rate
Low
High
P = 0.014
Time (months)
Number at risk
Low 28 8 7 4 3 0
High 28 16 10 4 1 1
CDYL gene
P = 0.58
Number at risk
Low 28 14 12 5 1 0
High 28 10 5 3 3 1
K
J82
circCDYL KD
Upregulated
EPITHELIAL MESEN-
CHYMALTRANSITION
Downregulated
INTERFERON ALPHA RESPONSE
E2F TARGETS
INTERFERON GAMMA RESPONSE
G2M CHECKPOINT
MYC TARGETS V1
-Log10(p-value)
IGF2BP1 KD
Downregulated
MYC TARGETS V1
E2F TARGETS
G2M CHECKPOINT
MTORC1 SIGNALING
-Log10(p-value)
IGF2BP2 KD
Upregulated
TNFA SIGNALING VIA NFKB
INTERFERON ALPHA RESPONSE
INTERFERON GAMMA RESPONSE
-Log10(p-value)
P
circRBM33
Overall survival
P = 0.0058
Number at risk
Low 28 17 10 1 0
High 28 21 5 2 0
RBM33 gene
P = 0.24
Number at risk
Low 28 18 7 1 0
High 28 20 8 2 0
circZKSCAN1
P = 0.02
Number at risk
Low 28 19 8 2 0
High 28 19 7 1 0
ZKSCAN1 gene
P = 0.39
Number at risk
Low 28 19 10 2 0
High 28 19 5 1 0
L
J82
UMUC3
Expression (FPKM)
GAPDH
IGF2BP1
IGF2BP2
circCDYL
Q
Total circRNA expression
P = 0.026
Number at risk
Low 28 18 10 1 0
High 28 20 5 2 0
Total gene expression
P = 0.14
Number at risk
Low 28 18 8 1 0
High 28 20 7 2 0
M
J82
P = 0.2
MYC expression (normalized counts)
circCDYL KD
scr
N
J82
P = 8e-04
TP53 expression (normalized counts)
circCDYL KD
scr

Fig. S6: CircCDYL interacts with RBPs in bladder cancer and abundance is associated with overall survival
A) Validation of IP by western blot. Overexpression of Twin-Strep-tagged RBPs for 48 hours followed by IP and RNA purification. Experiments are performed in the bladder cancer cell line FL3. IN = Input, FT = Flow through, IP = IP of RBP. **B+C)** CircCDYL (B) and *CDYL* (C) transcript expression levels relative to β-actin expression levels for IP and input in FL3. GFP was used as control. **D)** Expression of circCDYL and the corresponding linear transcript in eleven bladder cancer cell lines. UMUC3 and J82 were chosen for knockdown experiments based on circCDYL expression levels and cell line stability. **E)** Western blots of IGF2BP1, IGF2BP2, and GRWD1 knockdown in UMUC3 and J82 cells. **F)** Normalized expression levels of circCDYL and the *CDYL* gene upon circCDYL KD in UMUC3 and J82 cells. Expression is normalized to *GAPDH* and plotted relative to control (scr samples). P-values obtained by T-test. **G)** Differential expression analyses of mRNAs upon circCDYL KD in J82 (top) and UMUC3 (bottom). The log2 fold changes (circCDYL KD vs scr) are plotted against the negative log10(P-values). Colors indicate if genes are significantly down- (red) or upregulated (green) or not significantly differentially expressed (Not DE, grey) after Benjamini-Hochberg correction, FDR < 0.1. Vertical lines indicate a log2FC > 1 or < -1. **H)** Overlap of differentially expressed genes upon circCDYL KD in J82 and UMUC3 cells. P-value obtained by Fisher's Exact Test. **I)** Overlap of genes affected by GRWD1 KD in HepG2, UMUC3, and J82 cells. P-values obtained by Fisher's Exact Test. **J)** Overlap of genes affected by circCDYL KD, and IGF2BP1 and IGF2BP2 KD, respectively, in J82. P-values obtained by Fisher's Exact Test. **K)** Gene set enrichment analysis of 50 hallmarks of cancer upon circCDYL KD (top), IGF2BP1 KD (middle), and IGF2BP2 KD (bottom) in J82 cells. **L)** Expression of the exon that constitutes circCDYL (chr6:4891946-4892613) and the mRNAs of its interacting RBPs, *IGF2BP1* and *IGF2BP2*, in UMUC3 and J82 cells. *GAPDH* is used as reference for a highly expressed gene. **M+N)** Expression of *MYC* (M) and *TP53* (N) upon circCDYL KD in J82. P-values obtained by Wald Test. **O)** Kaplan-Meier recurrence-free survival plots for circCDYL and *CDYL* host gene. Median expression used as cutoff; circCDYL = 0.125 RPM and *CDYL* = 21.4 FPKM. P-values obtained by Log-Rank Test. **P)** Kaplan-Meier overall survival plots for circRBM33, *RBM33* host gene, circZKSCAN1, and *ZKSCAN1* host gene. Median expression used as cutoff; circRBM33 = 0.0618 RPM, *RBM33* = 21.3 FPKM, circZKSCAN1 = 0.0994 RPM, and *ZKSCAN1* = 17.8 FPKM. P-values obtained by Log-Rank Test. **Q)** Kaplan-Meier overall survival plots for total circRNA and gene expression. Median expression used as cutoff; circRNAs = 19.5 RPM and genes = 366,000 FPKM. P-values obtained by Log-Rank Test.
